# The Effect of Antimicrobial Treatment upon the Gill Bacteriome of Atlantic Salmon (*Salmo salar* L.) and Progression of Amoebic Gill Disease (AGD) In Vivo

**DOI:** 10.3390/microorganisms9050987

**Published:** 2021-05-02

**Authors:** Joel Slinger, Mark B. Adams, Chris N. Stratford, Megan Rigby, James W. Wynne

**Affiliations:** 1 CSIRO Agriculture and Food, Bribie Island Research Centre, Woorim, QLD 4507, Australia; chris.stratford@csiro.au; 2 Institute of Marine and Antarctic Studies, University of Tasmania, Launceston, TAS 7250, Australia; Mark.Adams@utas.edu.au; 3 CSIRO Agriculture and Food, Castray Esplanade, Hobart, TAS 7004, Australia; megan.rigby@csiro.au (M.R.); james.wynne@csiro.au (J.W.W.)

**Keywords:** aquaculture, salmon, AGD, antimicrobial, amoeba, gill, microbiome, perurans

## Abstract

Branchial surfaces of finfish species contain a microbial layer rich in commensal bacteria which can provide protection through competitive colonization and production of antimicrobial products. Upon disturbance or compromise, pathogenic microbiota may opportunistically infiltrate this protective barrier and initiate disease. Amoebic gill disease (AGD) is a globally significant health condition affecting salmonid mariculture. The current study examined whether altering the diversity and/or abundance of branchial bacteria could influence the development of experimentally induced AGD. Here, we challenged Atlantic salmon (*Salmo salar*) with *Neoparamoeba perurans* in a number of scenarios where the bacterial community on the gill was altered or in a state of instability. Administration of oxytetracycline (in-feed) and chloramine-T (immersion bath) significantly altered the bacterial load and diversity of bacterial taxa upon the gill surface, and shifted the community profile appreciably. AGD severity was marginally higher in fish previously subjected to chloramine-T treatment following 21 days post-challenge. This research suggests that AGD progression and severity was not clearly linked to specific bacterial taxa present in these systems. However, we identified AGD associated taxa including known pathogenic genus (*Aliivibrio*, *Tenacibaculum* and *Pseudomonas*) which increased in abundance as AGD progressed. Elucidation of a potential role for these bacterial taxa in AGD development is warranted.

## 1. Introduction

Teleost fish mucosa is a functionally important tissue constructed of macromolecules and polymers containing numerous enzymes and protective peptides [1,2]. The mucosa forms a structural medium that facilitates colonization of beneficial microbiota which play a key role in the health and function of the animal [3,4]. The bacterial community which colonize the mucosal layer contains both transient and resident taxa which utilize available resources [5] and perform key roles such as competitive exclusion or inhibition of unwanted pathogens [6,7,8,9].

Dysbiosis is a community level imbalance of microbial taxa, typically characterized by a disturbance or perturbation [10,11]. Environmental stressors or disease can lead to a dysbiosis of mucosal bacterial communities in an aquatic setting [12]. For example, rapid temperature reduction and air exposure applied to the late egg developmental stages significantly affected the gut and skin community of larval Atlantic salmon (*Salmo salar*) [13]. Similarly, transfer of Atlantic salmon smolt from freshwater to seawater caused an appreciable transition of the microbiota occupying the skin mucus [14]. Significant loss of bacterial richness and a destabilization of the skin community composition was reported from Atlantic salmon infected with sea lice [15]. *Aeromonas salmonicida* dominated the intestinal microbiota in furunculosis affected largemouth bronze gudgeon (*Coreius guichenoti*, exhibiting a significant dysbiosis compared to unaffected fish [16]. A largely unexplored area of microbial dysbiosis is the susceptibility of external barriers to pathogen outbreaks following bacterial dysbioses. Disease treatment or prevention via antimicrobial compounds is often crucial to mitigate stock losses in the event of bacterial or fungal infections, including prominent salmonid diseases such as sea lice (*Lepeophtheirus salmonis*), yellow mouth (*Tenacibaculum* spp.) and bacterial gill disease (*Flavobacterium* spp.) [17,18,19]. Typically, these treatments are ‘broad spectrum’ in nature, and can contribute to imbalances of the microbial consortia [2].

Amoebic gill disease (AGD) is a proliferative gill condition predominantly affecting salmonid mariculture. The causative agent, *Neoparamoeba perurans* is a free-living marine amoeba species which attaches to gill lamellae eliciting focal necrosis, oedema, inflammation and hyperplasia of the gill epithelium [20,21,22,23,24,25] leading to physiological disturbance and mortality if untreated [26,27,28,29,30]. *N. perurans* is ubiquitously distributed throughout many salmon production areas [31,32], comprising part of the microbial community within the external milieu alongside numerous other marine microbes (e.g., viruses, fungi, bacteria, other protozoa). The external surfaces of finfish species such as Atlantic salmon are in constant contact with these pathogens and the potential threat they pose.

While the primary pathogenic role of *N. perurans* in AGD has been unequivocally confirmed via Koch’s postulates [23], knowledge gaps exist regarding potential relationships with other microbes. Initiation of AGD occurs via adherence of *N. perurans* to the gill mucosa where commensal bacteria are present [22,33,34]. Given that amoebae can utilize bacteria as a feed source or coexist in a symbiotic arrangement it remains possible that bacterial taxa may have a role in the progression of AGD [35,36]. A limited number of previous studies have suggested that particular bacteria are associated with amoebic branchialitis and/or may affect the onset and severity of this condition. *Pseudomonas* sp. were observed within and around trophozoites of *Paramoeba* sp. isolated from AGD affected Atlantic salmon with small round bacteria observed in histological gill sections from the corresponding fish [22]. A culture-independent study of gill bacteria [37] demonstrated a small number of AGD affected fish in both field and laboratory scenarios were dominated by a phylotype assigned to *Psychroserpens* sp. The authors proposed this species was a potential opportunistic pathogen associated with AGD. A subsequent culture-dependent study observed the genera *Winogradskyella* and *Staphylococcus* in association with AGD affected Atlantic salmon [38] proposing a similar link or association of this bacterium with AGD. A follow-up study found a higher percentage of lesion affected gill filaments in Atlantic salmon following colonization *Winogradskyella* sp. and challenge with *Neoparamoeba sp.* [33]. Recently, it was confirmed that the bacterial community associated with AGD affected gill lesions can be dominated by pathogenic species such as *Tenacibaculum dicentrarchi* [36]. The pathogenicity of other disease-causing amoeba species can be affected by bacterial presence. For example, *Entamoeba histolytica,* responsible for mammalian intestinal enteritis were co-cultured with known pathogenic bacteria increasing the rate of adhesion and cytopathic effect to host cell lines [39]. Amoeba keratitis of the human eye caused by numerous *Acanthamoeba sp.* has been strongly linked to several bacterial co-factors, including *Corynebacterium xerosis* [40]. The timing and nature of interactions from pathogenic amoebae species as an opportunistic or synergistic process, and the subsequent impact on the commensal resident microbiota is not yet fully understood.

Identification of bacterial taxa that colonize the gills during an amoebic infection may provide further understanding of interactions between amoebae and bacterial species, and whether these interactions play a role in onset and progression of AGD. Therefore, the aim of the present study was to investigate the impact upon experimentally induced AGD progression by altering the bacterial load upon the gills prior to infection by *N. perurans*. We also examined whether bacterial community structure and diversity is altered by experimental infection and disease caused by *N. perurans*.

## 2. Materials and Methods

All animal activities relating to fish use in this trial were approved by the CSIRO QLD Animal Ethics Committee under the permit numbers CQAEC 2017–35 and CQAEC 2018–18.

### 2.1. Fish Source and Husbandry

Atlantic salmon fingerlings (all female, diploid) obtained from the Rookwood Road hatchery in Ranelagh, Tasmania at approximately 8 g mean weight were transferred to a freshwater recirculating aquaculture system (RAS) (5000 L) at the Bribie Island Research Centre. Fry were ongrown for approximately 8 months, before being exposed to 24 h light (3200 lumen) for a period of 5 weeks, after which the water salinity was raised from 3 ppt to approximately 36 ppt. Fish were acclimated at this salinity for approximately 4 weeks prior to the trial commencing. Water temperature was held at 15 °C ± 0.5, dissolved oxygen at 90–110% sat, TA-N < 0.50 mg·L^−1^, and salinity 35–36 ppt.

### 2.2. Experimental Design, Procedures and Maintenance

Ninety fish were randomly selected by dip-net and transferred to an independent 500 L tank and fed oxytetracycline hydrochloride (OTC) administered in-feed for 10 days (CCD, Tamworth, NSW, Australia). Feed pellets (3 mm Spectra, Skretting Pty Ltd., Cambridge, TAS, Australia) were vacuum coated at 350 P.S.I for 5 min with a 2% fish oil-based emulsion containing 79 mg·kg^−1^ OTC powder for every kg of feed, such that a 1% bodyweight ration would equate to a dosage of 79 mg·kg^−1^ of fish biomass. OTC coated pellets were stored at −20 °C, with the required ration taken from the freezer to be loaded into the autofeeders each morning.

### 2.3. Pre-Challenge with Antimicrobial Treatments

**Figure 1:**
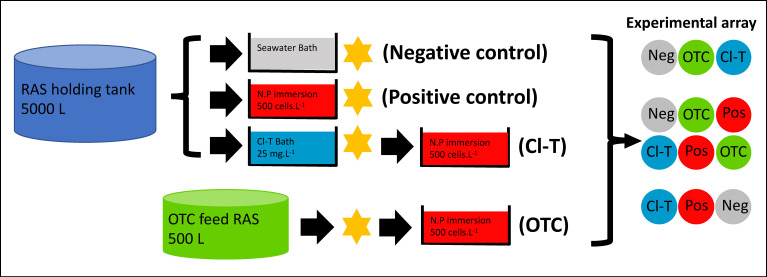
Flow diagram of experimental treatment preparation used in this study, showing treatment or challenge steps prior to stocking into experimental array (each treatment group was replicated three times). Star shape depicts when initial (Time 0) gill mucus sampling of 5 fish per tank (15 fish per treatment group) was completed.

At the beginning of the trial period Atlantic salmon post-smolt (350 ± 1 g) were anaesthetized using 17 mg·L^−1^ AQUI-S^®^ (Aqui-S Ltd., Lower Hutt, NZ, New Zealand), and exposed to a combination of bathing steps as depicted in Figure 1, dependent on the experimental treatment (*n* = 30 fish per bath replicate; 90 fish total per treatment).

Fish selected for the chloramine-trihydrate (Cl-T) therapeutic bath treatment were netted out into a disinfected plastic tub, containing a homogenized solution with 300 L of filtered seawater (15 °C) and 7.5 g of chloramine-trihydrate powder (Sigma-Aldrich, St. Louis, MO, USA) under constant aeration (~90–100% Sat.). This nominal dose of 25 mg·L^−1^ was verified by measuring (total chlorine – free chlorine) × 3.97 (Y.S.I 9500 photometer), and applied for a period of 1 h, as per FDA recommendations for therapeutic application in aquaculture.

After antimicrobial treatment, fish were stocked into an array of 12 identical 500 L tanks (*n* = 25 per tank). Array tanks assigned to the AGD affected (positive control, Cl-T) and AGD naïve (negative control) treatments were offered a feed ration of 1% bodyweight daily (3 mm Skretting Spectrum pellet) via autofeeders (Arvotec wolf controller, Arvotec-Oy, Huutokoski, Finland). Fish from the OTC fed holding RAS were also stocked into the flow through system and maintained the OTC treatment dose of 79 mg·kg^−1^ for the experimental duration (21 days). Experimental tanks were supplied with flowthrough seawater (~6 L·min^−1^) that was filtered (20 µm), ozonated (100 gO_3_·h^−1^), UV sterilized (80 mJ·cm^2^) and chilled to ~15 °C. Fish were monitored via constant data logging of water temperature and dissolved oxygen (Oxyguard Pacific, Farum, Denmark) and photoperiod was maintained at 12^L^:12^D^. Daily maintenance was carried out in the form of observing fish behavior for overt signs of AGD including listlessness and excessive opercular movement, as well as cleaning tank systems and collection of any uneaten feed at the conclusion of the autofeeder activity period. This collected feed was retained into a mesh sieve, where a pellet count could be taken to ensure normal feed rates occurred.

### 2.4. Sampling Strategy

#### 2.4.1. Gill Mucus Collection

Sampling of fish gill mucus was carried out directly following antimicrobial treatment (Figure 1), at the 11 dpi midpoint sample (5 fish per tank), and again at 21 dpi (15 fish per tank). At each sampling timepoint five individual fish from each tank (15 fish per treatment) were randomly dip netted from the tank and euthanized (by immersion in 100 mg·L^−1^ AQUI-S^®^). A sample of mucus was taken by swabbing the surface of all anterior and posterior hemibranchs from the entire left-hand side of the gill basket (8 surfaces). This was achieved by rotating a sterile cotton swab (Westlabs, Ballarat, VIC, Australia) three times on each of the hemibranch surfaces. Swabs were then transferred to a 1.5 mL screw cap tube containing 1 mL of RNAlater solution, and stored at 4 °C for 24 h before being frozen and stored at −80 °C until further processing.

#### 2.4.2. Tank Water Samples

Representative water samples were obtained from all water sources used during trial setup in triplicate, along with each experimental tank at the 11- and 21-day post inoculation (dpi) sampling points. Briefly, ~700 mL of water was collected in triplicate disinfected HDPE containers by placing the container approximately 10 cm subsurface and opening the lid. Each of these samples were then filtered across a 0.22 µm Sterivex filter membrane (Millipore, Burlington, MA, USA) using a peristaltic pump unit (Lachat Instruments, Milwaukee, WI, USA), and disinfected tubing with luerlok fittings. The filter chamber was then flooded with approximately 3.5 mL of RNAlater solution and stored at 4 °C prior to DNA extraction.

### 2.5. DNA Extraction and Purification

#### 2.5.1. Gill Mucus

All gill mucus samples underwent DNA extraction using the DNeasy PowerSoil kit (Qiagen, Hilden, Germany). RNAlater preserved swabs were placed into a Tissue Lyser (Qiagen, Hilden, Germany) for 10 min at a frequency setting of 15.0 Hz before pulse centrifuging of each individual tube. The swabs were removed using a sterile forceps, taking care not to cross-contaminate samples, and placed into a labelled 2 mL tube. The remaining RNAlater was then spun down at 17,000× *g* for 10 min in order to form a visible pellet. RNAlater was then pipetted to waste, taking care not to dislodge the pellet. Both the pellet and the swab were stored at −80 °C until processing. To process, 60 µL of Solution C1 was added to the PowerBead tube, mixed by pipetting up and down, then 200 µL was removed and used to collect the thawed pellet, with all liquid returned to the PowerBead tube along with the corresponding swab. The PowerBead tube containing the pellet and swab was then sharply tapped upside down on the benchtop to ensure the beads moved freely around the swab. Samples were then vortexed horizontally using a vortex adapter tube holder at maximum speed for 20 min. Extraction steps were completed according to the manufacturer’s instructions, with an elution volume of 50 uL and genomic DNA was assessed for yield and quality using a Nanodrop ND-1000 spectrophotometer (Life Technologies, Carlsbad, CA, USA). Samples were stored at −20 °C until downstream use.

#### 2.5.2. Tank Water

Bacterial DNA was extracted from 0.22 um Sterivex (Millipore, Burlington, MA, USA) water filter units. Filter samples were extracted using the DNeasy PowerWater Sterivex kit (Qiagen, Hilden, Germany), as per manufacturers protocols. Genomic DNA quality and concentration was verified using Nanodrop ND-1000 spectrophotometer (Life Technologies, Carlsbad, CA, USA). DNA from triplicate pooled samples were combined prior to storage. Samples were stored at −20 °C until sequencing.

### 2.6. Challenge with Neoparamoeba Perurans and AGD Assessment

An immersion bath containing *Neoparamoeba perurans* trophozoites in seawater was used to challenge fish in the AGD positive control, Cl-T and OTC groups (Figure 1). A dedicated ‘constant infection tank’ (CIT) consisting of a 2000 L RAS containing Atlantic salmon smolt to passage wild-type *N. perurans* was used for this experiment. Firstly, water from this CIT system (1 L) was collected using sterile 50 mL tubes and concentrated down by centrifuge at 4000× *g* to a final volume of 10 mL. Counts of this subsample on a hemocytometer (*n* = 10) were then used to enumerate the concentration of amoebae cells per liter within the CIT. After estimating the amoebae load within the infection system, a sufficient volume of well-homogenized water was transferred to a disinfected 500 L tub, and made up to 100 L with filtered seawater to achieve a cell concentration of 500 cells·L^−1^. Fish were netted into this bath and maintained for a period of 1 h, with supplemental aeration provided. The AGD naïve treatment group (negative control) underwent a sham immersion bath containing only filtered seawater. All fish were hand netted into the experimental array after this process, and the *N. perurans* bath setup step was repeated with each replicate to account for amoebae cells lost from the bath via adherence to the gill.

#### 2.6.1. Gill Score Assessment

Following euthanasia but prior to sample collection the gross gill score was recorded for each sampled fish. AGD gill scoring was performed as described by [41], where all 16 arches are visually assessed for white multifocal mucoid patches. A score between 0 (no visible AGD) and 5 (severe AGD) was then assigned to each individual fish to give a gill index per tank and treatment.

#### 2.6.2. Gill Histopathology

The gill basket from each fish was excised using sterile micro scissors and placed into a specimen jar containing seawater Davidson’s fixative, where each holobranch was dissected individually and all 16 arch surfaces were photographed using a lightbox and SLR camera (Canon EOS 7D), before transfer to 70% EtOH after 48 h. Subsequently, the third arch from the right-hand side (R3) was excised from the gill basket, and routinely processed, infiltrated and embedded in paraffin. Samples were sectioned (5 μm) from the anterior hemibranch surface using a Microm microtome (Thermo Scientific, Waltham, MA, USA) and placed onto glass slides. The slides were then stained (H&E), cover-slipped and examined under a Leica DM1000 light microscope (Leica Microsystems, Wetzlar, Germany). The hemibranch section was assessed for the proportion of filaments with hyperplastic gill lesions and the percentage of lesions with *N. perurans* present.

#### 2.6.3. Quantitative PCR Assay

DNA from gill mucus samples (obtained in 2.5.1) were analyzed using a TaqMan^®^ qPCR targeting the 18 S rRNA gene sequence of *N. perurans* generating an amplicon of 70 bp (Table S1). The salmon elongation factor gene (Ef1α) described in [42] was used as the reference gene in this assay, amplifying a 66 bp fragment. Each real-time PCR reaction mixture contained 4 μL template, 5 μL TaqMan^®^ Fast Advanced Master Mix (Applied Biosystems) with a final reaction volume of 10 μL. The thermal profile of the real-time PCR program consisted of 2 min at 50 °C, 2 min at 95 °C, followed by 45 cycles of 1 s at 95 °C and 20 s at 56 °C in an QuantStudio™ Real-Time PCR instrument (Applied Biosystems, Foster City, CA, USA). Each plate included a *N. perurans* positive and negative control, as well as a PCR ‘no template’ control in triplicate for both the target and reference gene. Samples were run in triplicate for *N. perurans* and duplicate for salmonid elongation factor-1α (Ef1α). Analysis of the real-time data involved setting the threshold across all plates for *N. perurans* and Ef1α at 0.1 and 0.04, respectively. Quantitative PCR data was assessed using the delta-delta Ct (2^–∆∆Ct^) method derived by [43]. Data were estimated by comparing the ratio of ∆Ct of the gene of interest (*N. perurans* 18 S)-∆Ct of the housekeeping gene (salmon Ef1α) for each gill swab sample. After this, ∆∆Ct was calculated by measuring the ∆Ct (treated fish sample)-∆Ct (untreated fish mean) and the relative fold gene expression change was calculated by transforming data (2^–∆∆Ct^).

### 2.7. Gill Bacteriome Assessment

#### 2.7.1. Branchial Bacteria Counts

A gill mucus swab of the right-hand side anterior hemibranch (R1) was collected from 5 fish per tank (*n* = 15 per treatment) to estimate culturable bacterial loads on the gill. Samples were taken via a sterile cotton swab (Westlabs, Ballarat, VIC, Australia) of the hemibranch (three rotations along the length of the arch) and placing the swab into 1 mL of filtered, autoclaved seawater in a 1.5 mL tube. Each tube was then agitated via vortex for 15 s, before a 500 L aliquot was pipetted onto individual Petri-film^®^ aerobic count films (3M, Saint Paul, MN, USA) and incubated at 35 ± 0.1 °C for 48 h. After incubation discrete colonies were visually counted within the film grid area and recorded (data were calculated as CFU·mL^−1^).

#### 2.7.2. 16 S rRNA Amplicon Sequencing

DNA obtained from gill mucus (2.5.1) and tank water (2.6.1) underwent amplicon sequencing, targeting the V1–V3 hypervariable region of the 16 S rRNA gene. This was prepared via a “2-step” PCR submission process, using the Illumina recommended adapter-fused overhangs applied to the V1–V3 amplicon primers (bold) as shown in Table S2. A total of 25 cycles were performed with normalized DNA at an average of 10 ng·µL^−1^. Samples in this study included a mock positive control (ZymoBIOMICS Microbial Community Standard, Zymo Research), and two negative controls (blank swab process control and blank DNA extraction laboratory control). Sequencing was performed at the University of New South Wales, (Ramaciotti Center for Genomics Sydney, Australia) via an Illumina Miseq platform with 300 base pair (bp) paired end reads.

### 2.8. Bioinformatics Pipeline

Raw Illumina amplicon sequencing data files were processed using the open-source software pipeline “Quantitative Insights into Microbial Ecology 2” QIIME2 [44]. Paired end sequences from the forward and reverse reads were merged for each sample and were denoised using the q2-dada2 plugin [45] with default parameters. Quality control including chimeric sequence removal from the dataset was completed during dada2 processing, along with subsequent removal of host DNA and exclusion of chloroplast and mitochondrial sequences. Amplicon Sequence Variants (ASV’s) were classified taxonomically using the classify-sklearn method in the QIIME2 q2-feature-classifier plugin using default parameters [46]. The SILVA 16 S rRNA 99% taxonomy database release 132, [47], was used as reference sequences for taxonomic classification.

### 2.9. Statistical Analysis

All statistics were performed in R version 3.6.1 [48], with QIIME2 artefact files imported using the Qiime2R package (https://github.com/jbisanz/qiime2R, accessed on 5 December 2020). For all statistical analyses, the significant *p*-value was <0.05, except where an adjusted significance is stated. In the amplicon data, obvious contaminant artefact present in the negative control sequences was identified and subsetted from biological samples via the Decontam package [49]. Samples were rarefied using R package QsRutils [50] performed on a maximum subsampling depth of 13,460 sequences per sample (Figure S1). Using the Phyloseq R package [51] alpha diversities were calculated based on observed ASVs, Shannon diversity and Faith’s phylogenetic distance metrics, and the differences between groups were analyzed using the non-parametric Kruskal–Wallis and Wilcoxon rank-sum tests. Beta-diversity comparisons were made via NMDS using Bray Curtis pairwise distances. Differences between groups was analyzed using PERMANOVA testing from the pairwise Adonis package [52]. Differential abundance testing was completed using microbiomeSeq package found in [53], where log-fold change of taxa prevalence at the genus level were compared between groups referenced against the negative control. Genera were considered significant at an adjusted *p*-value (*padj)* <0.01.

Count plate data (bacterial load) were expressed as CFU·mL^−1^ and a log transformation performed. The log-fold CFU data was assessed using a general linear model to test treatment by timepoint. The subsequent significant interaction was further substantiated using a one-way ANOVA and Tukey PSD post hoc testing (*padj* < 0.05). Gill score data (median) was compared using a non-parametric Kruskal–Wallis test, and using a Wilcoxon rank sum test to assess pairwise Treatment and Timepoint interactions. Quantitative PCR data were analyzed as log-fold change between treatment groups of the gene of interest (*N. perurans* 18 S) after normalization against the internal control (salmon Ef1α), log transformed and assessed using two-way ANOVA with treatment and timepoint as factors (*padj* < 0.05). Histology data for proportion of affected filaments and % of lesions colonized by amoebae was arcsine transformed (to account for non-normality, determined by Shapiro–Wilk testing) prior to statistical analysis. Data were assessed by one-way ANOVA, with subsequent pairwise comparison using a Tukey’s HSD test. All figures were produced using the R package ggplot2 [54].

## 3. Results

At the trial commencement, two mortalities occurred in the Cl-T treatment group, post amoebae exposure (identified as moribund on transfer to experimental tank, and humanely euthanized). Upon further inspection a lower jaw deformity and shortened opercula was identified in both fish. Due to the significant reduction in respiratory efficiency of jaw/opercula deformed fish, it was deduced during necropsy that these individuals may have succumbed to respiratory stress.

### 3.1. Onset and Progression of AGD after Challenge with N. perurans

**Figure 2:**
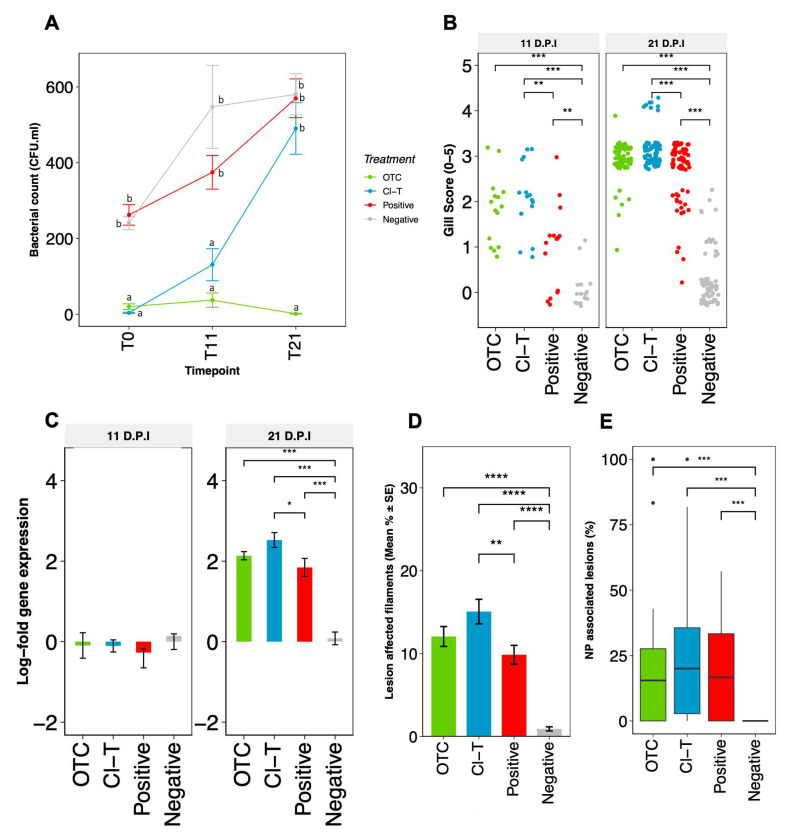
(**A**)-Bacterial counts ± SE obtained from gill mucus swabs derived from the RH1 anterior hemibranch surface. CFU·mL data was log-transformed for statistical analysis; letters indicate significantly different subset groups between all treatments and timepoints (padj < 0.05). (**B**)-Frequency dotplot of visual gill scores obtained from 11 and 21 dpi, with statistical comparison of treatment groups via nonparametric Kruskal Wallis, pairwise statistical differences are presented with asterisks (*p* < 0.01 = **, *p* < 0.001 = ***). (**C**)-Relative fold change ± SE (in log2 scale) of *N. perurans* 18 S gene expression as a function of the reference housekeeping gene (Ef1α) measured by RT-qPCR during amoebic challenge period (11 and 21 dpi). Each bar represents the mean for each experimental replicate (*n* = 15), statistical differences are presented with asterisks (*p* < 0.05 = *, *p* < 0.001 = ***). (**D**)-Histopathological quantitation (*n* = 30 per treatment) depicting the percentage of lesion affected filaments at 21 dpi with *N. perurans*. Statistical assessment is based on arcsine-transformed data, and presented with asterisks (*p* < 0.05 = *, *p* < 0.0001 = ****. (**E**)–Percentage of hyperplasic gill lesions or plaques colonized with amoebae trophozoites, outlying datapoints are represented by black dots, and pairwise statistical differences are presented with asterisks (*p* < 0.001 = ***).

Gross clinical signs of AGD including raised multifocal lesions on the gill surface were visually observed in AGD affected fish in all groups challenged with *N. perurans* (Figure 2B). Gill score post-inoculation increased from 11 dpi to 21 dpi in all AGD positive groups; OTC (+1.12), Cl-T (+1.05) and positive control (+1.75). At both timepoints, the gill scores of Cl-T and OTC treated fish were similar (*p* > 0.05), but the Cl-T and positive infection groups were significantly different (*p* < 0.05). Small mucoid patches were occasionally observed in unchallenged control fish at both timepoints. The gill index (0.30) remained equivocal at both timepoints but significantly different to all AGD positive groups (*p* < 0.001).

The log-fold delta-delta (∆∆Ct) change between expression of the 18 S *N. perurans* gene and the salmon Ef1α housekeeping gene demonstrated that the amoebic burden increased throughout the 21 dpi challenge period. When the target gene was standardized to the housekeeping gene of the mean AGD positive control ∆Ct these data suggest that the relative *N. perurans* burden was highest within the Cl-T treatment tanks, followed by the OTC and positive AGD groups at 21 dpi (Figure 2C). There was a statistical difference in qPCR data between the Cl-T and positive control fish (*padj* < 0.05), as well as between the negative non-AGD control and the three AGD exposed groups assessed (*padj* < 0.001 at 21 dpi, Figure 2C).

**Figure 3:**
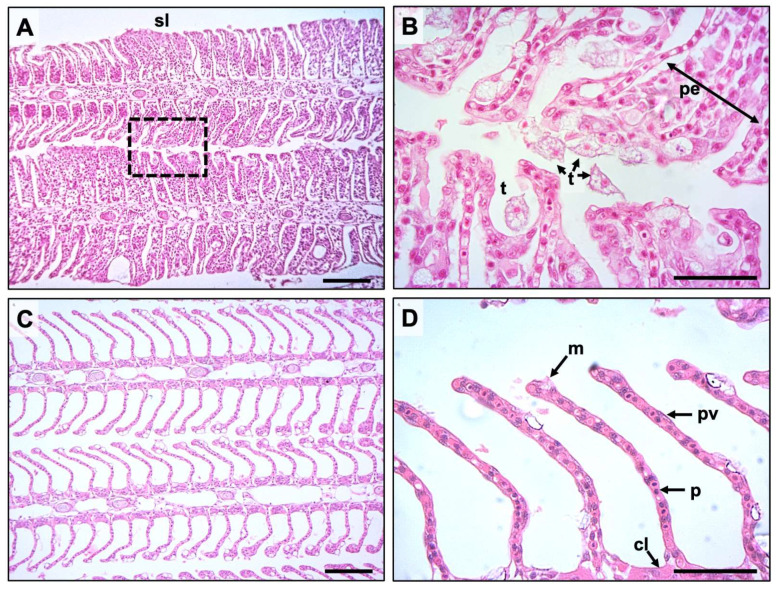
Representative histological sections of gill obtained from the experiment; (**A**) Gill lesion along both margins of primary lamellae, showing extensive fusion of secondary lamellae (sl), scale bar = 50 μm. (**B**) shows border inset from (**A**) at 400× magnification, demonstrating *N. perurans* trophozoites with clearly discernable nuclei (t) attached and adjacent to areas of proliferating epithelium (pe) scale bar = 100 μm. (**C**) negative control group, depicting anatomically normal gill morphology, scale bar = 50 μm. (**D**) Higher magnification of normal healthy gill (400×) showing typical cell types including mucus (m), pavement (pv), pillar (p) and chloride (cl) cells, scale bar = 100 μm.

At 21 dpi, pathological observations showed typical AGD lesions characterized by multifocal epithelial hyperplasia, lamellae fusion, interlamellar vesical formation and oedema (Figure 3A,B). *N. perurans* trophozoites with visible cell nuclei were also observed in aggregation along lesion margins (Figure 3B). Gills from unchallenged fish appeared largely normal (Figure 3C,D). Small lymphocytic nodules were occasionally observed (1–3 interlamellar units) in all groups. AGD affected gill filaments in the *N. perurans* challenged fish was highest in the Cl-T treated fish (15.06% SE ± 1.48), followed by OTC (12.05% SE ± 1.19) and positive (9.85% SE ± 1.35) groups. The percentage of lesion affected filaments in AGD exposed groups were significantly higher than unchallenged control (*p* < 0.001 Figure 2D). Amoeboid trophozoites were not observed in gill sections from unchallenged fish. Comparisons of AGD exposed fish identified that the Cl-T group was significantly higher than the positive group (*p* < 0.01) at 21 dpi. No significant interaction was observed between the OTC group and either the positive control or Cl-T groups. The proportion of lesions colonized by amoebae was slightly higher in Cl-T treated fish (Figure 2E), but there was no significant interaction between *N*. *perurans* exposed fish. The unchallenged (negative) control was significantly different from all AGD exposed groups (*p* < 0.001).

### 3.2. Gill Bacteriomic Profiles

#### 3.2.1. Following Antibacterial Treatment

Cultivable bacterial colony counts were significantly reduced in Cl-T directly post treatment, and in OTC throughout the experimental duration (Figure 2A). Samples from the positive and negative treatment groups were not significantly different from each other, but did increase over time from the 0, 11 and 21 dpi measures. The Cl-T group was significantly lower than the positive and negative groups in the initial timepoint (F = 7.112, df = 2,6, *p* < 0.001), but was not significantly different at the 21 dpi timepoint.

#### 3.2.2. Concurrent to *Neoparamoeba Perurans* Challenge

**Figure 4:**
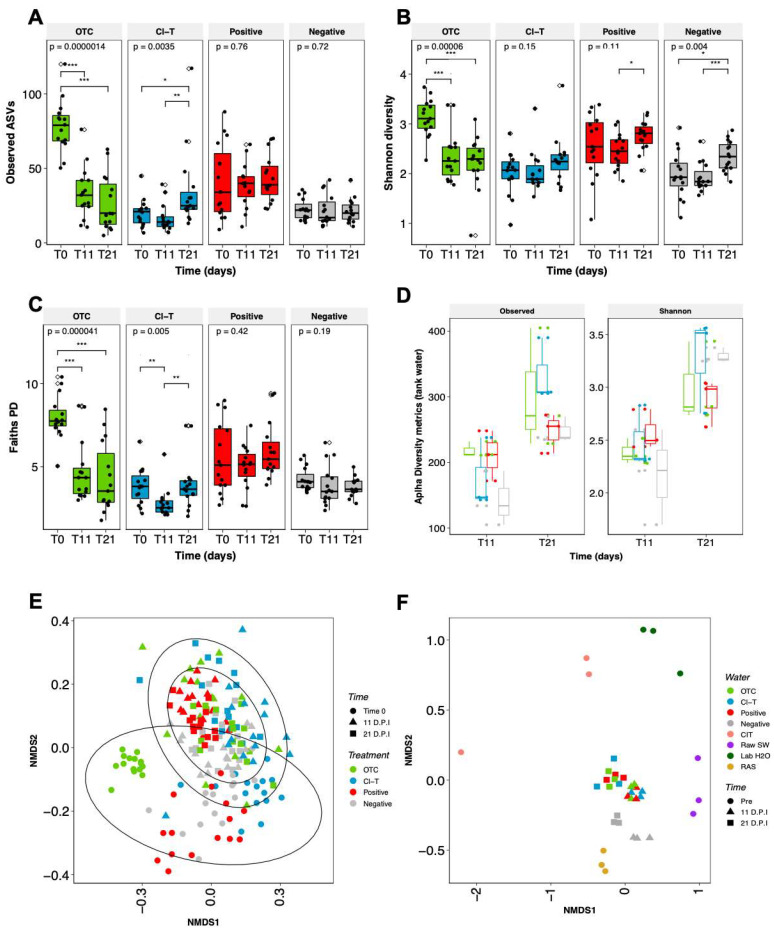
Alpha richness (**A**) expressed as Observed ASVs, and community diversity metrics (**B**,**C**) Shannon index and Faiths PD for gill mucus communities, along with Observed ASVs and Shannon index recorded in trial tank water (**D**). Black dots represent each individual sample point, unfilled diamond shape indicates outliers. P = global significance (Kruskal Wallis), pairwise significance determined by a Wilcoxon test with *p* < 0.05, *p* < 0.01 and *p* < 0.001 represented by *, **, and ***. Beta diversity visualized through Bray Curtis NMDS plots for (**E**) fish gill mucus and (**F**) holding tanks/water sources used in the trial.

After processing the *16 S rRNA* gene V1–V3 region sequencing data using QIIME2, an ASV table with 7515 assigned taxa was generated in 218 samples. Bacterial community richness was highest in the OTC treatment fish group at the initial timepoint of the experiment (Figure 4A). This was significantly different from the 11 dpi and 21 dpi timepoints for this treatment (KW, *p* < 0.001). The Observed ASVs in all other treatments slightly increased from T0 to 21 dpi, with the Cl-T increasing significantly between 11 dpi and 21 (Figure 4A, KW, *p* < 0.01). In parallel to the observed richness, Shannon diversity (Figure 4B) in the OTC group was highest at the commencement of the trial, decreasing sharply after this time. Shannon diversity in Cl-T, positive and negative treatment groups stabilized between T0 and 11 dpi, before increasing at 21 dpi. While the Shannon index did increase over time in the negative treatment, it remained largely static in terms of phylogenetic diversity (Faiths PD; Figure 4C). The AGD positive treatment group increased in phylogenetic diversity over the trial period although this was not significant. The OTC treatment decreased significantly between T0 and both 11 dpi and 21 dpi, whilst the Cl-T group decreased significantly at the mid-point (11 dpi; *p* < 0.01), but was largely unchanged between T0 and 21 dpi (Figure 4C). Alpha diversity in tank water sampled at 11 dpi and 21 dpi showed a dramatic increase in both observed ASVs and species diversity over time. The Cl-T and OTC tanks represented the highest richness and diversity at the 21 dpi timepoint (Figure 4D).

Beta diversity was visualized using NMDS ordinations based on Bray Curtis pairwise distances for all fish gill mucus and rearing water samples. PERMANOVA analysis showed that there was a significant interaction between gill mucus communities at each experimental timepoint (*F* = 11.9, df = 2, *p* < 0.001) and antimicrobial treatment group (*F* = 6.8, df = 3, *p* < 0.001). An interaction effect between the two factors, Treatment * Time was also observed (*F* = 5.29, df = 6, *p* < 0.001; Figure 4E). Pairwise Adonis revealed that there was a high degree of separation between treatment groups, with all treatment groups significantly different at time 0 (*p* < 0.001). As the trial progressed the fish gill community tended to converge closer. At day 11 positive and OTC groups were not significantly different, but the Cl-T treated fish were significantly different from all other groups at this timepoint (*p* < 0.001). At day 21, the Cl-T and positive groups were not significantly different, however both groups were significantly different to OTC treated fish (to Cl-T; *p* < 0.01, positive; *p* < 0.001). The negative control group remained significantly distinct from all other groups when compared at both 11 dpi (*p* < 0.001), and 21 dpi (to OTC; *p* < 0.05, Cl-T; *p* < 0.01, positive; *p* < 0.001).

Ordinations of each sample of tank or source water (Figure 4F) were visualized throughout the trial period. Trial array tanks showed a high rate of consistency between AGD positive treatments, and shifted together between the 11 and 21 dpi timepoint. These treatments were not significantly different from one another within each timepoint, but timepoint groupings (11 dpi vs. 21 dpi) were deemed significantly disparate when analyzed using PERMANOVA (*F* =14.2, df = 6, *p* < 0.001). The negative control tanks remained closely aligned, but were more similar to the RAS source tank where fish were smoltified, and were significantly different from all other trial treatment groups (*p* < 0.05). Filtered lab seawater, raw seawater (unfiltered, undisinfected) and CIT (amoebae inoculum water) represented more distinct communities.

Taxonomic assignment at the phylum level revealed 19 distinct taxa from fish gill mucus. The most dominant taxa in this study were *Proteobacteria* (39.01%), *Verrucomicrobia* (29.85%) and *Bacteriodetes* (16.1%) (Figure S2A). Initial timepoints for all treatment groups besides the positive control tended to have a much lower proportion of *Verrucomicrobia,* which dramatically increased post handling to 21 dpi. The OTC treatment had a much lower proportion of *Actinobacteria* comparative to other treatment groups at the initial sampling event, and subsequently a much higher proportion of *Bacteriodetes* within the 15 samples. Between all treatments the most dominant taxa were *Proteobacteria*, *Verrucomicrobia*, *Bacteriodetes* and *Actinobacteria* at 21 dpi. Tank water and experimental source water showed a strong presence of *Bacteriodetes* and *Proteobacteria*, with the two phyla composing a majority of the community with a small proportion of *Firmicutes* and *Actinobacteria* among other more cryptic taxa (Figure S2B). Source water provided to the experimental array appeared to carry a much higher proportion of *Verrucomicrobia* in contrast, with a small amount of this taxa found in both the CIT inoculum as well as the raw unfiltered seawater pumped onshore from the estuary.

**Figure 5:**
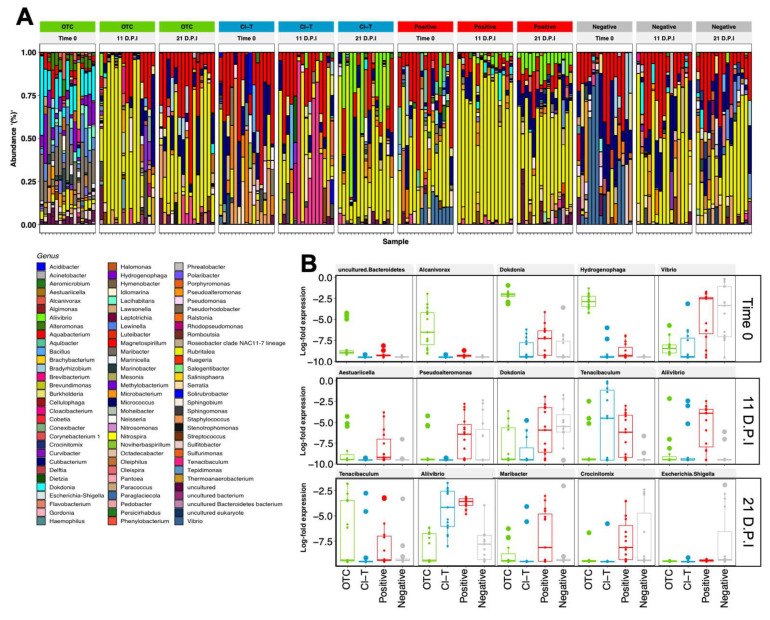
(**A**)-Relative abundance of the top 93 genera assigned to fish gill mucus samples at three timepoints. Each bar represents one fish gill sample for the respective treatment group. (**B**)-Differentially expressed (*padj* < 0.01) taxa between treatment groups within each of the three measured timepoints. Top 5 taxa are ranked based on ASV importance (ascending in significance level) and expressed as log2 fold differences. The AGD positive treatments showed higher abundance of *Aliivibrio* at 11 dpi and higher *Tenacibaculum* at both 11 dpi and 21.

The ASV assignments demonstrated 356 genera found in fish gill communities, with *Rubritalea* (33.12%), *Aquabacterium* (15.24%), *Cutibacterium* (8.83%) and *Staphylococcus* (2.95%) the most prevalent (Figure 5A). The *Rubritalea* taxon increased at each timepoint for all 4 treatment groups, after being largely absent in initially stocked fish. Gill mucus samples had high consistency between independent replicate tanks for each treatment group, but distinct treatment-based differences (PERMANOVA, *p* < 0.001). *Aquabacterium* was a dominant taxon within the gill surface mucosa in OTC, Cl-T and negative treatments, and was particularly abundant at the 11 dpi timepoint. At the 21 dpi timepoint *Aliivibrio* was prominent in the Cl-T and positive control treatments, but was not identified in the OTC group. In all AGD exposed treatments, the genus *Tenacibaculum* was present in low abundance, and increased towards 21 dpi. The exception was the Cl-T treatment at the 11 dpi timepoint, which had a much higher relative abundance of this taxa. Genus assignments for water samples (Figure S3) were characterized by *Dokdonia* (10.37%), an ASV assigned as ‘uncultured’ (9.68%—identified as family *Saprospiracea* and *Caldilineaceae* at family level classification), *Rubritalea* (8.36%) and *Aliivibrio* (7.85%) (Figure S3). The RAS holding tank had a small proportion of *Dokdonia*, along with the experimental tanks from all treatment groups. After having high proportions in the 11 dpi sample point, this taxon decreased by 21 dpi. In all AGD positive treatments the identified *Saprospiracea* taxa increased in prevalence at 21 dpi, along with *Aliivibrio*, which also mirrored the gill mucus samples at 21 dpi.

Differentially abundant taxa were calculated for fish gill mucus samples within each treatment group when referenced against the negative control group, with the 5 most differentially abundant taxa (*padj* < 0.01) plotted in Figure 5B. The Time 0 sample expressed higher proportions of *Dokdonia* and *Hydrogenophaga* in the OTC treatment. *Vibrio* was most prevalent at this timepoint in the negative and positive groups. The 11 dpi sample point had significant increases in *Tenacibaculum* in Cl-T and positive treatments, as well as *Aliivibrio* in the latter. At 21 dpi, *Tenacibaculum* and *Aliivibrio* were expressed at a higher proportion (*p* < 0.001) than the negative treatment. A much higher proportion of *Escherichia Shigella* was observed in non-AGD affected fish at this timepoint.

**Figure 6:**
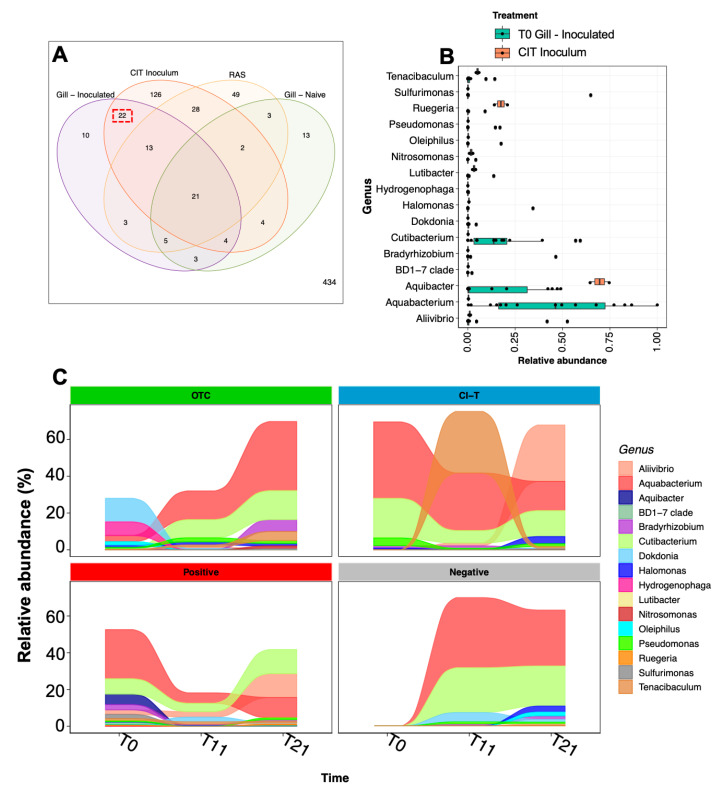
Bacterial taxa associated with AGD sources. (**A**) Venn diagram of shared ASV sequences between the AGD inoculum (CIT), and post inoculated gill (Time 0), naïve gill from the same cohort (Time 0) and the holding tank RAS, showing 22 ASVs shared between AGD inoculum and inoculated gills (Red dashed box). (**B**) Shows relative abundance of genus assigned to shared ASVs in the AGD inoculum and post challenged fish at Time 0. (**C**) Demonstrates the longitudinal relative abundance of representative key genus assignments over the course of the trial. Taxa including *Aliivibrio*, *Tenacibaculum*, *Cutibacterium* and *Bradyrhizobium* increase toward 21 dpi in AGD positive treatments.

During the initial inoculation process, 22 unique ASVs were identified on the gill surface of post-inoculated fish, which were common only to the CIT inoculum water and not present on the gills of unchallenged fish or in the holding tank they originated from (Figure 6A). These ASV sequences corresponded to 16 genus assignments (Figure 6B), which were dominated by *Aquabacterium*, *Aquibacter* and *Cutibacterium*. Longitudinal relative abundance of the 16 genera revealed that these three taxa along with *Allivibrio, Tenacibaculum* and *Pseudomonas* were prominently featured in all groups (Figure 6C).

## 4. Discussion

The mucosal bacterial community plays a key role in health and vitality of fish, yet we have limited understanding of the effect that commensal bacterial imbalance plays in disease susceptibility. Here, we compared the progression of AGD between groups of Atlantic salmon with modulated gill bacterial communities, and assessed the role of bacterial taxa in AGD development.

Antimicrobial treatment was effective in altering the gill mucosal bacterial community load and diversity. Colony counts from culture plates inoculated with gill mucus indicated that viable cultivable bacteria numbers were reduced following antimicrobial treatment (both OTC and Cl-T). This was also reflected by disparate bacteriomic data observed after antimicrobial treatment, indicating that the gill mucus bacteria had been effectively altered. Previous studies using oxidative compounds have also demonstrated effective removal of culturable bacterial flora from the gill surface with products such as potassium permanganate [55]. *Gambusia affinis* immersed in the antibiotic Rifampicin also demonstrated a significant decrease in bacterial load on the skin surface [56] lasting for 1.6 days.

After successful reduction of the bacterial load on the gills we next challenged fish with virulent *N. perurans* and compared the progression of AGD. The severity of AGD via presumptive gill score at 11 dpi (mid-point) of the trial demonstrated the gill score index was significantly higher in Cl-T comparative to the positive AGD control group. This result may indicate a more rapid progression of AGD at that stage of the challenge. In contrast, the severity of AGD in fish treated with OTC was not significantly different to untreated AGD-affected fish, despite their significant reduction in bacterial load on the gills. It is possible that initial oxidative bath treatment may have led to increased AGD susceptibility. The physiological impacts of oxidative therapeutics such as Cl-T and hydrogen peroxide on the gills include congestion of the filaments, oedema and epithelial lifting [19,57,58,59]. Denuding the mucosal bound layer may hinder any innate protective functions. While it was demonstrated that mechanical damage to the gill epithelium did not lead to increased AGD progression [60], limited data exists regarding pre-exposure to oxidative chemicals prior to *N. perurans* challenge. It is possible the downstream impact of stress from such oxidative treatment may compromise the innate immunity of the fish, facilitating favorable colonization of *N. perurans* and faster progression of AGD. Chloramine-T has been identified as a non-specific immune suppressant in rainbow trout, where fish exposed to 5 ppm immersion for 3 h demonstrated a decrease in plasma lysozyme and serum bactericidal activity [61]. Other oxidative topical treatments such as hydrogen peroxide and peracetic acid also induce both physiological and oxidative stress on Atlantic salmon [62,63].

Bacterial count data at 11 dpi showed the gills of fish bathed in Cl-T were rapidly recolonized with bacteria, although the OTC group remained at negligible levels. Taxonomically, genus level bacteria between treated and untreated groups were vastly different, indicating that antimicrobial action had led to a community level imbalance. It is likely that the bacterial taxa susceptible to OTC and Cl-T may have been removed, and subsequently replaced on the gill surface with compound-resistant species which were not able to be cultured on count plates and thus not able to be quantified using the count plate method. We observed high levels of *Tenacibaculum* in the Cl-T group at the 11 dpi. Similarly, [64] observed detrimental impacts post Cl-T treatment in rainbow trout, where the skin microbial layer was left infection prone and colonized by secondary opportunists (*Tenacibaculum* and *Pseudomonas)*. It remains possible that the increased incidence of known pathogenic bacteria such as *Tenacibaculum* in the Cl-T group may have also contributed to any increase in the onset of AGD. Several publications have examined the incidental co-abundance of *Tenacibaculum* and amoebic branchialitis, and surmised that severity may increase in the presence of both pathogens [36,65,66]. Evidence for bacterial co-factor virulence in other ectoparasitic conditions exist, including Ich (*Ichthyophthirius multifiliis*) in the presence of *Aeromonas hydrophila* [67,68], and fish lice (*Argulus coregoni*) when co-infected with *F. columnare* [69].

Progression of AGD at the 21 dpi sampling point was verified by an increase in mean gill score and increased *N. perurans* 18S gene abundance. These were highest in Cl-T treated fish, and significantly different to the untreated positive control group. Concomitantly, branchial histopathology of fish challenged with *N. perurans* indicated that the Cl-T bathed group also had the highest percentage of lesion affected filaments, differing significantly to the AGD positive control. Taken together, results of presumptive gill scoring, parasite qPCR assays, and histopathology suggested that AGD had advanced marginally further within the group of fish treated with Cl-T prior to challenge. In similar scenarios, potassium permanganate (KMnO_4_) treatment of channel catfish (*Ictalurus puntactus*) caused bacterial diversity imbalances of the skin, which was further exacerbated when challenged with *Flavobacterium columnare* [70]. Contrastingly, the effect on bacterial diversity was not as impactful for fish which were not KMnO_4_ treated pre-challenge. Other studies have postulated that fish may be more susceptible to secondary pathogens as a result of a prior dysbiosis [71,72]. Further research investigating the mechanisms of such disease susceptibility is required to improve our understanding of how bacterial dysbiosis affects disease pathogenesis.

Viable bacterial counts increased in all experimental groups (besides OTC) longitudinally with the Cl-T, positive and negative control groups finishing 21 dpi at similar CFU·mL^−1^ loads. The slight but consistent increase in both the untreated AGD challenged and AGD naive fish may point to the tank environment taking some time to establish after transition from RAS to flow-through water sources. The cultivable bacterial load of healthy rainbow trout (*Oncorhynchus mykiss*) gills was reported to be around 4.95 × 10^3^ CFU·mL^−1^ [73]. These data were obtained from gill material of 7 holobranchs and appear to agree with the current study compared to a single hemibranch surface. Bacterial sequence data was largely in agreeance with aerobic count plate data. Observed ASVs and Shannon diversity for the Cl-T, positive and negative fish peaked at the 21 dpi sample point, possibly indicating that the gill mucosa was accumulating bacterial taxa through the trial duration. These data are supported by the alpha diversity values recorded for tank water, where diversity and richness increased from 11 dpi to 21 dpi. In contrast, diversity and richness of gill mucus in the OTC treated group decreased over time. Reduction in commensal bacterial diversity and richness post-antibiotic treatment has been well documented in a range of aquatic species. For example, Atlantic salmon [74] when treated with in-feed OTC showed a dramatic reduction in diversity of intestinal bacteria. Both [75] and [76] demonstrated that the microbial perturbation of such antibiotic usage can last upwards of 18 days. In this study we did not observe a significant dysbiosis over the progression of AGD at 21 dpi, compared to other studies which have observed such perturbations in health-affected fish [72,77]. However, the infection load of *N. perurans* and consequential development of gill lesions in the current study suggested a light to moderate disease response. Previously we had demonstrated that more advanced AGD contributes to a lower mucus bacteria diversity [36,78].

Interestingly, the presence of both *Winogradskyella* and *Staphlococcus* in water samples from AGD affected tanks agree with previous work [33]. This study cultured colonies from gill mucus swabs to obtain several bacterial isolates which may have been present in concert with AGD affected Atlantic salmon, but absent in fish naïve to the condition. Historically, numerous challenge methodologies have been employed for inducing AGD experimentally, including cohabitation of trojan AGD affected fish to a naïve cohort [79,80], or by excising the gill basket of AGD affected individuals to harvest gill-attached trophozoites (wild-type) [60,81,82]. The current study employed an immersion challenge using water from a dedicated AGD constant infection tank (CIT) which passaged AGD affected hosts to maintain *N. perurans* load, as per previous studies in this facility [83,84]. Due to difficulty or impracticality cultivating axenic *N. perurans* for fish challenge [85,86,87], all in vitro and in vivo methods appear to be concomitantly linked with bacterial growth. It is logical to assume that because of the xenic nature of *N. perurans* exposure in all inoculation scenarios, bacterial components are also simultaneously exposed to the gill. This study characterized the bacterial biomass associated with the immersion challenge inoculum, which may be linked to the ecology of *N. perurans*. There were 22 distinct ASVs identified that colonized salmon gill surfaces immediately following inoculation of the tanks with water containing *N. perurans.* The 17 genus level assignments were prominently featured on the gills in most groups. The bacterial taxa which were significantly differentially expressed in AGD positive groups to 21 dpi included known pathogenic taxa, *Aliivibrio*, *Pseudomonas* and *Tenacibaculum*. These specific genera have been identified in high abundances in several fish disease settings including gut enteritis of yellowtail kingfish (*Seriola lalandi*), as well as winter ulcer disease and sea louse infestations in Atlantic salmon [15,76,88]. The limitation of having bacterial rich amoebae for AGD research means that to comprehensively examine the impact of bacterial co-factors an experimental protocol incorporating axenic trophozoites would need to be completed. A vaguely assigned ASV (uncultured) at genus level made up a large component of the tank community in all AGD positive groups at the latter stages of the trial. This classification was investigated via family level assignments to be predominately composed mainly of *Saprospiracea* and to lesser extent *Caldilineaceae*, identified using the NCBI BLAST tool (Seq. ID AB625329.1 and JF514230.1). Although the exact species is unknown, the groups of *Saprospiracea* related taxa are known for hydrolysis and utilization of complex carbon sources [89].

In conclusion the present study suggests that reducing gill bacteria that were sensitive to orally administered OTC did not significantly affect the progression of experimentally induced AGD. However, bath treatment with chloramine-T prior to amoebic challenge led to marginal advancement of AGD in salmon smolt. We demonstrate that AGD developed with different levels of bacterial dysbiosis, and progressed concurrently with increased colonization of potential secondary pathogenic bacterial taxa including *Aliivibrio* and *Tenacibaculum*, coinciding with the peak of the AGD severity observed in this study. To examine this further, the functional role or relationship of these bacterial taxa in AGD development warrants further enquiry.

## Data Availability

Raw sequence data and sample metadata from this study is stored in the NCBI SRA repository under the accession number PRJNA718152.

## Supplementary Materials

The following are available online at https://www.mdpi.com/article/10.3390/microorganisms9050987/s1, Table S1. *Neoparamoeba perurans* and salmon elongation factor taqman quantitative PCR validation of primer and probe concentrations. Table S2. Validation of Illumina overhang adapter and 27F-519R primer sequences for 16S rRNA V1-3 amplicon sequencing. Figure S1. Alpha rarefaction curve for biological samples that underwent 16S rRNA microbiome sequencing. Figure S2. Relative abundance of phylum level assignments of bacteria from (A) salmon gill mucus and (B) culture water sources. Figure S3. Relative abundance of top 91 genus assigned to culture water sources in the experiment, and tank water in experimental tank units.
