# Supplementary material for: The Effect of Antimicrobial Treatment upon the Gill Bacteriome of Atlantic Salmon (Salmo salar L.) and Progression of Amoebic Gill Disease (AGD) In Vivo
Source: Microorganisms. 2021 May 2;9(5):987. doi: 10.3390/microorganisms9050987 (PMC8147422; doi:10.3390/microorganisms9050987)
Supplement: Supplementary file 1 [file microorganisms-09-00987-s001.zip › microorganisms-1185443-supplementary.pdf]

Supplementary:

Table S1. Primer sequence and concentration for qPCR assay target genes, *Neoparamoeba perurans* and salmon elongation factor 1 alpha (ELF)

Gene	Primer	Sequence (5'-3')	Reaction Conc. (nM)	Amplicon Length (bp)	Reference
N. perurans	Forward	AAAAGACCATGCGATTCGTAAAGT	300	70	[1,3]
	Reverse	CATTCTTTTCGGAGAGTGGAAATT	900		
	Probe	6FAM-ATCATGATTCACCATATGTT-MGB	200		
Ef1α	Forward	GGCCAGATCTCCCAGGGCTAT	900	66	[2]
	Reverse	TGAACTTGCAGGCGATGTGA	900		
	Probe	NED-CCTGTGCTGGATTGCCATACTG-MGB	250		

Table S2. Primer sequences for the 2-step PCR preparation for amplifying the V1-V3 region within the 16S rRNA gene

Forward primer 5′-3′ (“27F-adapt”)		
Illumina overhang adapter	27F	Refs
TCGTCGGCAGCGTCAGATGTGTATAAGAGACAG	AGAGTTTGATYMTGGCTCAG	[4,5]
Reverse primer 5′-3′ (“519R-adapt”)		
Illumina overhang adapter	519R	Refs
GTCTCGTGGGCTCGGAGATGTGTATAAGAGACAG	GWATTACCGCGGCKGCTG	[6,7]

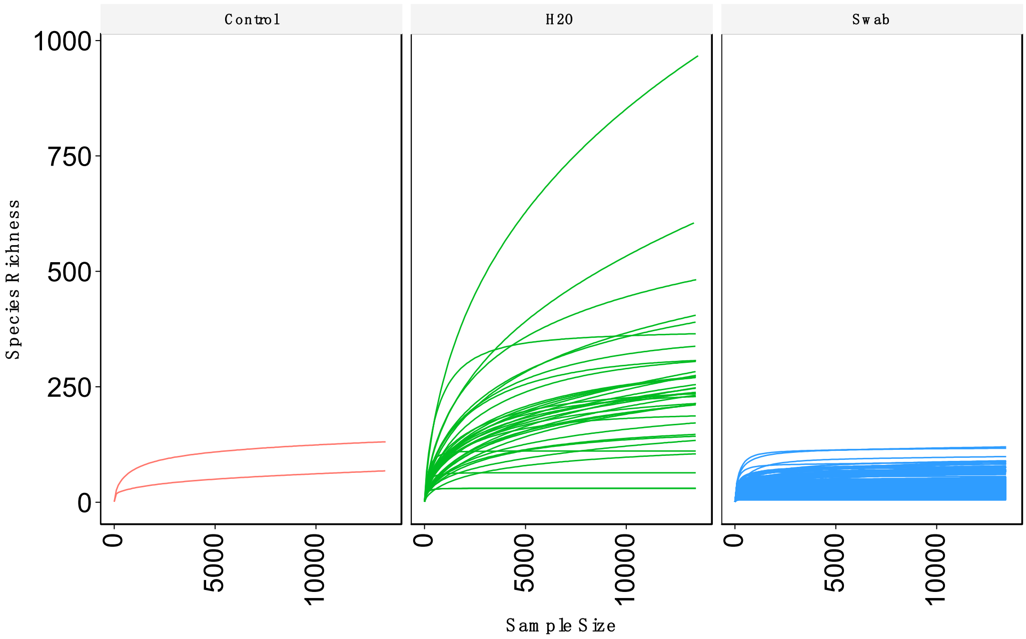

Figure S1. Alpha rarefaction curve from biological samples, depicting species richness (Observed ASVs) against sample size (sequence reads). Figure panels are faceted to sequencing controls, water samples and gill mucus swabs.

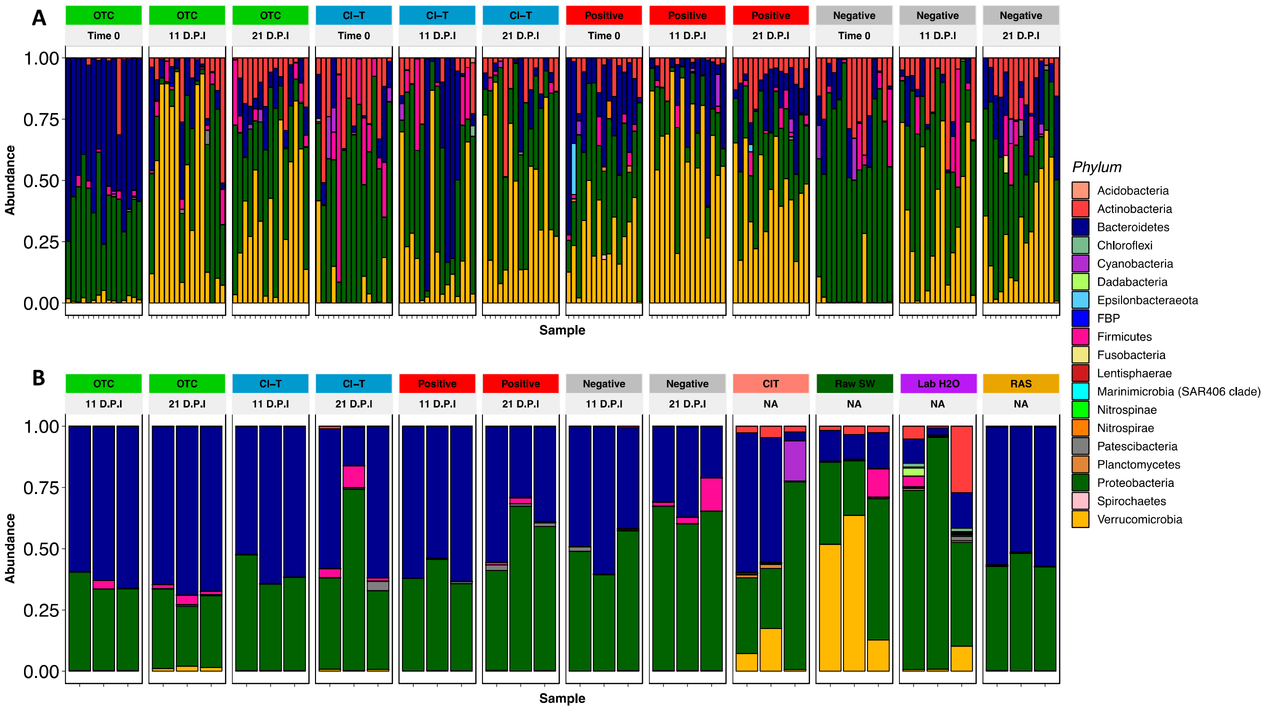

Figure S2. Relative abundance of phylum level ASV assignments for (A) fish gill mucus and (B) source water samples taken within the experimental trial. Data shows that fish gills were rapidly colonized by *Verrucomicrobia* post stocking in all treatments. Source lab water taken at the beginning of the trial also shared this ASV

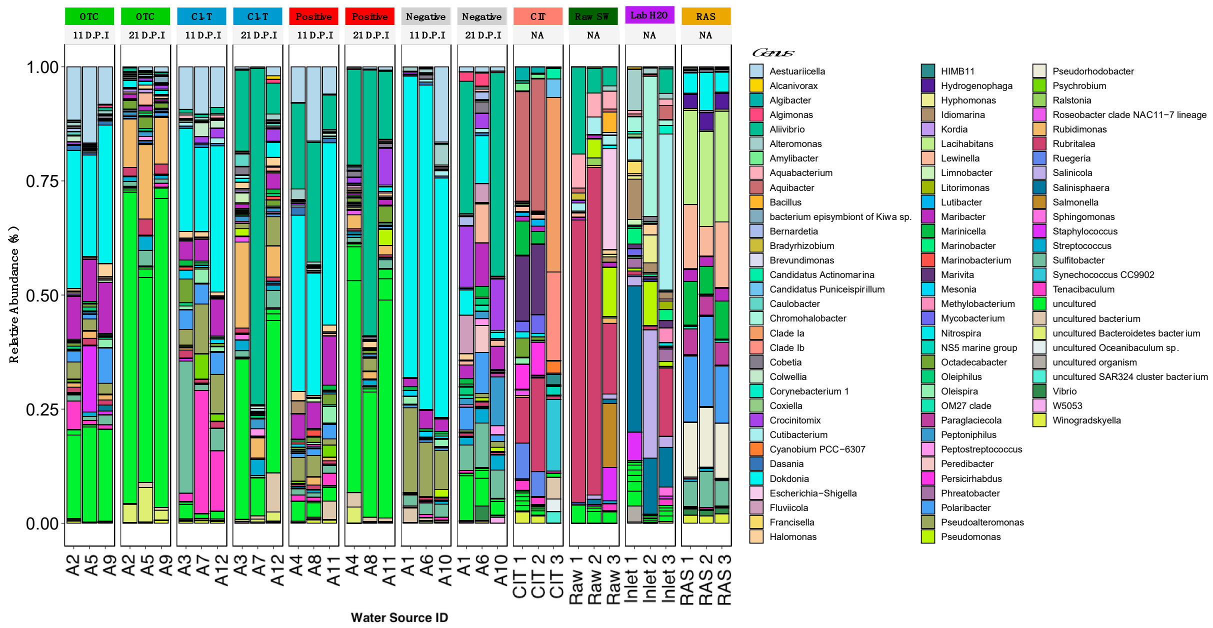

Figure S3. Top 91 genus assigned to various water sources used in the experimental trial, and tank water from experimental units. Each bar represents one pooled sample (3 x 700 mL water from each source). Data demonstrates that the assigned genus "uncultured" (Family *Saprospiracea* & *Caldilineaceae*) were prominent in all AGD affected treatments at 21 dpi.

References

1. Downes, J.; Henshilwood, K.; Collins, E.; Ryan, A.; O'Connor, I.; Rodger, H.; MacCarthy, E.; Ruane, N. A longitudinal study of amoebic gill disease on a marine Atlantic salmon farm utilising a real-time PCR assay for the detection of *Neoparamoeba perurans*. *Aquac. Environ. Interact.* **2015**, *7*, 239–251.

2. Bruno, D.; Collet, B.; Turnbull, A.; Kilburn, R.; Walker, A.; Pendrey, D.; McIntosh, A.; Urquhart, K.; Taylor, G. Evaluation and development of diagnostic methods for *Renibacterium salmoninarum* causing bacterial kidney disease (BKD) in the UK. *Aquaculture* **2007**, *269*, 114–122.
3. Downes, J.K.; Rigby, M.L.; Taylor, R.S.; Maynard, B.T.; MacCarthy, E.; O'Connor, I.; Marcos-Lopez, M.; Rodger, H.D.; Collins, E.; Ruane, N.M.; et al. Evaluation of non-destructive molecular diagnostics for the detection of *Neoparamoeba perurans*. *Front. Mar. Sci.* **2017**, *4*, 61.
4. Lane, D.J.; Pace, B.; Olsen, G.J.; Stahl, D.A.; Sogin, M.L.; Pace, N.R. Rapid determination of 16S ribosomal RNA sequences for phylogenetic analyses. *Proc. Natl. Acad. Sci. USA* **1985**, *82*, 6955–6959.
5. Zheng, W.; Tsompana, M.; Ruscitto, A.; Sharma, A.; Genco, R.; Sun, Y.; Buck, M.J. An accurate and efficient experimental approach for characterization of the complex oral microbiota. *Microbiome* **2015**, *3*, 48.
6. Lane, D.J. *rRNA Sequencing*; John Wiley & Sons: Hoboken, NJ, USA, 1991.
7. O'Farrell, H.E.; Shaw, J.G.; Goh, F.; Bowman, R.V.; Fong, K.M.; Krause, L.; Yang, I.A. Potential clinical utility of multiple target quantitative polymerase chain reaction (qPCR) array to detect microbial pathogens in patients with chronic obstructive pulmonary disease (COPD). *J. Thorac. Dis.* **2019**, *11*, S2254–S2265.
